# Ready to Use Therapeutic Foods (RUTF) improves undernutrition among ART-treated, HIV-positive children in Dar es Salaam, Tanzania

**DOI:** 10.1186/1475-2891-11-60

**Published:** 2012-08-29

**Authors:** Bruno F Sunguya, Krishna C Poudel, Linda B Mlunde, Keiko Otsuka, Junko Yasuoka, David P Urassa, Namala P Mkopi, Masamine Jimba

**Affiliations:** 1 Department of Community and Global Health, Graduate School of Medicine, The University of Tokyo, 7-3-1 Hongo, Bunkyo-ku, Tokyo 113-0033, Japan; 2 School of Community and Global Health, Claremont Graduate University, 18 East Via Verde Ste. 100, Claremont, CA 91773, USA; 3 School of Public Health and Social Sciences, Muhimbili University of Health and Allied Sciences, P.O Box 65489, Dar es Salaam, Tanzania; 4 Department of Pediatric and Child Health, Muhimbili National Hospital, P.O Box 65000, Dar es Salaam, Tanzania

**Keywords:** Undernutrition, Ready-to-use therapeutic food, HIV/AIDS, Tanzania

## Abstract

**Background:**

HIV/AIDS is associated with an increased burden of undernutrition among children even under antiretroviral therapy (ART). To treat undernutrition, WHO endorsed the use of Ready to Use Therapeutic Foods (RUTF) that can reduce case fatality and undernutrition among ART-naïve HIV-positive children. However, its effects are not studied among ART-treated, HIV-positive children. Therefore, we examined the association between RUTF use with underweight, wasting, and stunting statuses among ART-treated HIV-positive children in Dar es Salaam, Tanzania.

**Methods:**

This cross-sectional study was conducted from September-October 2010. The target population was 219 ART-treated, HIV-positive children and the same number of their caregivers. We used questionnaires to measure socio-economic factors, food security, RUTF-use, and ART-duration. Our outcome variables were underweight, wasting, and stunting statuses.

**Results:**

Of 219 ART-treated, HIV-positive children, 140 (63.9%) had received RUTF intervention prior to the interview. The percentages of underweight and wasting among non-RUTF-receivers were 12.4% and 16.5%; whereas those of RUTF-receivers were 3.0% (P = 0.006) and 2.8% (P = 0.001), respectively. RUTF-receivers were less likely to have underweight (Adjusted Odd Ratio (AOR) =0.19, CI: 0.04, 0.78), and wasting (AOR = 0.24, CI: 0.07, 0.81), compared to non RUTF-receivers. Among RUTF receivers, children treated for at least four months (n = 84) were less likely to have underweight (P = 0.049), wasting (P = 0.049) and stunting (P < 0.001).

**Conclusions:**

Among HIV-positive children under ART, the provision of RUTF for at least four months was associated with low proportions of undernutrition status. RUTF has a potential to improve undernutrition among HIV-positive children under ART in the clinical settings in Dar es Salaam, Tanzania.

## Background

Management of undernutrition among HIV-positive children is complicated. This is mostly due to a weakened immunity [1] which leads to a higher case fatality rate even under the standard treatment guidelines [2]. To improve and maintain better nutritional status, the World Health Organization (WHO) encouraged the use of Ready to Use Therapeutic Foods (RUTF) for community-based treatment of severe undernutrition [3,4]. RUTF is energy-dense lipid paste made of peanut butter, milk powder, oil, sugar, minerals, vitamin, and protein mix [5].

Among RUTF-treated children, severe undernutrition status can be improved among hospitalized antiretroviral therapy (ART) naïve HIV-positive children [6]. Compared with HIV-negative children, the case fatality rate is higher among hospitalized ART naïve HIV-positive children [7,8]; however, community-based RUTF treatment of the ART-naïve HIV-positive children, particularly with severe undernutrition without complications, is known to be associated with a better survival [9].

The combination of RUTF and ART treatments might improve undernutrition and reduce case fatality rates among HIV-positive children. This is because ART improves immunity, reduces energy loss, and may improve child’s appetite to food [10-12]. So far, only a handful of studies have evaluated the effectiveness of each intervention separately and only one has shown that the initiation of RUTF and ART may improve wasting status among HIV-positive children [13]. In this study, prompt initiation of ART among patients treated with RUTF was associated with weight gain. However, this study focused on wasting alone and the association of RUTF treatment duration and nutrition statuses were not examined.

Tanzania is one of the countries hardest hit by both HIV/AIDS and child undernutrition. In 2009, HIV prevalence was 5.9% in Tanzania [14]. In the general population, 47.8% of children are stunted, 24.5% are underweight, and more than 4.5% are wasted [15]. ART program has been expanded in Tanzania [14]. However, ART-treated, HIV-positive children continue to suffer from underweight and wasting [16]. For example, 22.1% and 13.6% of ART-treated, HIV-positive children were underweight and wasted, respectively, in Dar es Salaam [16].

To ameliorate such high undernutrition rate, Ministry of Health and other development partners established the RUTF intervention in selected HIV/AIDS Care and Treatment Centers (CTCs) as a pilot intervention in Tanzania. Although studies among ART naïve populations showed effectiveness of RUTF, we could not find similar studies among ART-treated children elsewhere. Furthermore, since the start of RUTF intervention as a pilot project in Tanzania, no studies have evaluated its efficacy among ART-treated HIV-positive children. Therefore, we conducted this study to examine the association of RUTF intervention with stunting, wasting, and underweight statuses among the ART-treated, HIV-positive children in Dar es Salaam, Tanzania.

## Methods

We conducted this cross sectional study in Dar es Salaam, where HIV prevalence was 8.9% in 2009. It accounts for about 300,000 people living with HIV/AIDS. The city has 44 HIV/AIDS care and treatment centers (CTCs) which provide ART programs to care about 4,000 children [14].

### Participants and selection criteria

Participants of this study included pairs of 219 under-five ART-treated, HIV-positive children and their caregivers. These children were attending CTCs that provide RUTF treatment to severe undernourished children. In Dar es Salaam, a total of six out of 44 CTCs provide RUTF to such children. We excluded the CTC of the Muhimbili National Hospital (MNH). MNH is the referral center for complicated cases from the Northern zone and beyond. Being the highest tertiary hospital, its CTC also cares for more complicated and severe cases from the outside of the region. As a result, we selected five CTCs: Amana, Temeke, Mwananyamala, IDC, and Pasada CTCs. Children under five years of age in the selected CTCs totaled 1719. Amana CTC registered 350 children, Temeke CTC 396, IDC 250, Pasada 373, and Mwananyamala 350.

Of the total, about 58% of the children enrolled in CTCs were on ART. We excluded children with missing medical and ART-related information and those whose parents did not consent to participate in the study. We selected each alternative participant of this study from the list of the eligible participants (n = 670). We used Epi-Info Version 6 (CDC, Atlanta, USA) to calculate the minimum required sample size. We estimated that the ratio of children exposed to RUTF to those who were not exposed to RUTF in the intervention clinics to be 2:1. The proportion of underweight among ART-treated HIV-positive children was considered at 13.6% [16]. We therefore estimated the minimum sample size at the power of 80 and with 95% CI to be 173 (115 RUTF treated and 53 RUTF naïve HIV-positive children under ART). In this study, we collected the data of 219 ART treated HIV-positive children (140 RUTF treated and 79 RUTF naïve HIV-positive children under ART).

We defined ART-treated HIV-positive children as children whose sero-status was confirmed to be positive using standard laboratory methods and were taking Highly Active Antiretroviral Therapy (HAART). According to the national guidelines for pediatric HIV/AIDS care in Tanzania, children are typically treated with a combination therapy consisting of three antiretroviral medicines, hence HAART [17].

### RUTF intervention

RUTF Plumpynut® is given to children with severe wasting (Weight-for-height Z-score (WHZ) < −3SD) or underweight (Weight-for-age Z-score (WAZ) < −3SD) or both severe wasting and underweight [18]. Such children are treated with the recommended dosage of 200 kilocalories per kilogram per day until they reach the target weight, which should be in 6 to 10 weeks [5]. RUTF is a community-based intervention given to undernourished children who have no other clinical complications [3,9,19]. In this study, 140 HIV ART-treated, HIV-positive children who fulfilled the criteria received RUTF.

### Measures

We measured children’s weight by two methods. For children who could not stand alone, we used a standardized hanging Salter scale® (UK) calibrated to 0.1 kg. For children who could stand alone we used a standardized Seka® digital scale (Brooklyn, USA). We measured height for the 24 months and older children [20] using a Seka® measuring rod calibrated to 0.5 cm. We used the same measure on a board for the children who were less than 24 months, and took their lengths in a recumbent position [20]. We converted height and weight into height-for-age z-score (HAZ), weight-for-age z-score (WAZ), and weight-for-height z-score (WHZ) [21] by the Epi-Info ENA Ver. 3.5.1, 2008 (CDC, Atlanta, Georgia, USA) software, using WHO reference values [22,23].

Low HAZ, WAZ, and WHZ are the measures of stunting, underweight, and wasting, respectively [24]. Stunting reflects a chronic failure to receive adequate quality and quantity of nutrition over a long period of time; it may also signify a chronic recurrent illness [25]. Stunting therefore may be reflected by failure to attain normal height for the age of a normal child [24,26]. Wasting in most cases signifies an acute or recent and severe process of weight loss, which is associated with acute starvation or severe disease with nutritional deficit [25]. Underweight is also a measure of acute undernutrition, but is not as robust as wasting in this regard, since the child may be underweight because of stunting, wasting or both [27].

According to the WHO Global Database on Child Growth and Malnutrition, an abnormal anthropometry is defined as a value of Z-score below −2 Standard Deviation (SD) or above +2SD. Z-scores less than -2SD are defined as moderate undernutrition, while less than -3SD are defined as severe undernutrition [28]. These cut-off points define the central 95% of the reference distribution as the normality range [28].

We adopted the socio-demographic variables pertaining to children and their caregivers from the Tanzania Demographic and Health Survey (TDHS), women and household questionnaires [15]. Information collected included education level, religion and marital status. We defined a caregiver as child’s caretaker, parent, or a guardian that is involved in child’s routine care and who accompanied the child to the clinic. Education was classified as either: low (up to primary school level) or high (higher than secondary school level). Religions were categorized into two major groups in Tanzania i.e. Christians and Muslims. Marital status was categorized into either currently married or not [15], caregivers who were divorced or widowed at the time of data collection were considered as not currently married.

We assessed food security by using the 6-item Household Food Security Scale (HFSS) [29]. HFSS has been used in various settings including the US [30], Bolivia, Burkina Faso, Philippines [31], and the Caribbean [32]. This scale is used to measure household food security by 12-month recall. Characterization is made based on the sum of affirmative responses; two or more affirmatives indicates ‘food insecurity’, while 5 or more affirmatives indicate ‘hunger’. In this study, Cronbach’s alpha for the HFSS was 0.72, with corrected item-total correlation ranging from 0.09 to 0.74.

We assessed economic status by using a Weighted Wealth Index incorporating household durable assets ownership such as paraffin lamp, television, radio, telephone, flat iron, refrigerator, bicycle, motor car, farm and electricity; housing and dwelling characteristics, such as main floor materials, house ownership, fuel for lighting and cooking, type of toilet, source of water, feeding characteristics, and household food satisfaction [15]. Then, we constructed dichotomous variables and conducted principle component analysis (PCA) to reduce 42 items to 22 (loaded as factor 1). We also used factor loadings as item weights. We totaled item weights to yield the wealth index for each household [33-35].

### Data collection

We hired five nurse counselors and trained them for one day on interviewing technique and questionnaire contents. The first author and trained research assistants pretested the questionnaire. To ensure good interviewing environment, we recruited all research assistants from the clinics and assigned them to conduct data collection. We collected data by face-to-face interview with caregivers and anthropometric measurements of the children attending the clinic between September and October 2010.

### Data analysis

We conducted descriptive analysis using Chi-square test and Independent sample T-tests to compare characteristics and nutrition status of children who were treated versus those who were not treated with RUTF. We conducted multivariate analysis to examine the effectiveness of RUTF on nutritional status of HIV positive children after adjusting other covariates and important confounders including ART treatment duration. Among RUTF treated children, we used bivariate analysis to evaluate the association of undernutrition and RUTF treatment duration; since the proportion of wasting and underweight was small for multivariate regression analysis. We set the statistical significance at *P-value* <0.05. We conducted analyses using PASW 18 (SPSS Inc., Chicago, Illinois, USA).

### Ethical consideration

We obtained ethical clearance from the Ethical Committees of the University of Tokyo and Muhimbili University of Health and Allied Sciences to conduct this study. We also obtained permission to conduct research from health departments and facilities used for the study. Participation was voluntary, and confidentiality was ensured. Caregivers gave the written informed consent before starting the interview.

## Results

### Descriptive results

Out of 219 HIV positive children recruited for this study, 140 (63.9%) had received the RUTF intervention. Children who received and those who did not receive the intervention did not have any significant difference in age, sex, orphan hood state, and birth weight (Table 1). Caregivers of children who received and those who did not receive the intervention were not different in their mean ages, education level, religion groups, employment and marital status. Households of children in the intervention group did not have differences in wealth index from those who were not in the intervention (P = 0.266). A total of 19 (13.6%) of RUTF treated compared to 13 (16.5%) of RUTF naïve children came from households with food insecurity. Hunger was prevalent in households of 45 (32.8%) RUTF receivers compared to 41 (46.8%) of RUTF naïve children, (P = 0.004). Children in the intervention group received RUTF at the mean duration of about four months.

**Table 1:** Descriptive analysis of the study participants (N = 219)

	**RUTF-treated (N = 140)**				**RUTF-naive (N = 79)**				
**Variable**	**N**	**Mean**	**%**	**SD**	**N**	**Mean**	**%**	**SD**	**P-Value**
**Age child** (Months)		37.2		15.6		36.1		17.3	0.648
**Sex**									
Male	81		57.9		42		53.2		0.502
Female	59		42.1		37		46.8		
**Orphan hood**									
Yes	90		64.3		41		51.9		0.099
No	50		35.7		38		48.1		
**Birth weight**									
≥ 2500 g	110		78.6		57		72.2		0.365
< 2500 g	30		21.4		22		27.8		
**Age caregiver** (Years)		34.2		9.4		33.8		8.2	0.753
**Caregiver's education**									
≤ Primary	113		80.7		67		84.8		0.564
≥ Secondary	27		19.3		12		15.2		
**Major religions**									
Muslim	88		63.3		52		65.8		0.822
Christian	51		36.7		27		34.2		
**Employment status**									
Unemployed	84		60.0		52		65.8		0.479
Employed	56		40.0		27		34.2		
**Marital status caregiver**									
Married	95		67.9		47		59.5		0.237
Not married	45		32.1		32		40.5		
**Wealth index (tercile)**									
High	36		25.7		16		20.3		0.266
Middle	54		38.6		26		32.9		
Low	50		35.7		37		46.8		
**Food security score**									
Secured	76		54.3		25		31.6		0.004
Insecure	19		13.6		13		16.5		
Hunger	45		32.1		41		51.9		
**WAZ (underweight)**									
> − 2SD*	136		97.2		68		86.1		0.006
> − 3SD**	2		1.4		8		10.1		
<−3SD***	2		1.4		3		2.3		
**WHZ (wasting)**									
> − 2SD*	136		97.2		191		83.5		0.001
> − 3SD**	1		0.7		18		11.4		
<−3SD***	3		2.1		4		5.1		
**HAZ (stunting)**									
> − 2SD*	98		70.0		43		54.4		0.066
> − 3SD**	20		14.3		16		20.3		
<−3SD***	22		15.7		20		25.3		

* Normal, ** Moderate, *** Severe.

### Nutrition status

Table 1 also shows the descriptive analysis of nutrition status. At the time of data collection, 97.2% of RUTF treated children had a normal weight-for-age compared to 86.1% of RUTF naïve children (P = 0.006). Compared to 83.5% of RUTF naïve children, 97.1% of RUTF treated children had a normal weight-for-height (P = 0.002). Though a statistically significant difference was not detected, RUTF treated children had a lower proportion of moderate to severe stunting compared to RUTF-naïve children (30.0% vs. 45.6%, P = 0.066).

### Effectiveness of RUTF on nutrition status of ART-treated HIV-positive children

Table 2 shows the results of adjusted analyses on effectiveness of RUTF. ART-treated HIV-positive children who received RUTF were less likely to have underweight status (AOR 0.19, 95% CI: 0.04, 0.78, P = 0.021) and wasting status (AOR = 0.24, 95% CI: 0.07, 0.81, P = 0.021) compared to RUTF naïve ART-treated HIV-positive children. No significant difference was found on stunting risk between children who received RUTF and those who did not receive it in the adjusted analysis (AOR = 0.80, 95% CI: 0.41, 1.57, P = 0.511). Other factors associated with risk of underweight included male sex, lower economical status, food insecurity, and lower birth weight. Besides the RUTF intervention, wasting was also associated with lower birth weight, low economic status, and food insecurity. In this study, stunting was associated with only household’s hunger.

**Table 2:** Multiple logistic regression; effectiveness of RUTF among HIV positive children

**Variable**	**WAZ < −2SD**				**WHZ < −2SD**				**HAZ < −2SD**			
	**AOR**	**95% C.I. for AOR**		**P-Value**	**AOR**	**95% C.I. for AOR**		**P-Value**	**AOR**	**95% C.I. for AOR**		**P-Value**
		**Lower**	**Upper**			**Lower**	**Upper**			**Lower**	**Upper**	
**RUTF**												
No	1.00				1.00				1.00			
Yes	0.19	0.04	0.78	0.021	0.24	0.07	0.81	0.021	0.80	0.41	1.57	0.511
**Age (Ref <38 months)**	0.96	0.21	4.38	0.960	0.44	0.12	1.66	0.225	0.72	0.35	1.50	0.379
**Sex**												
Male	1.00				1.00				1.00			
Female	0.14	0.03	0.70	0.016	0.96	0.30	3.04	0.944	1.44	0.75	2.77	0.271
**Birth weight**												
≥ 2500	1.00				1.00				1.00			
< 2500	4.77	1.16	19.67	0.031	4.88	1.52	15.63	0.008	1.66	0.80	3.45	0.176
**Caregiver's education**												
≤ Primary	1.00				1.00				1.00			
≥ Secondary	0.11	0.01	1.28	0.077	0.41	0.08	2.22	0.300	0.84	0.36	1.97	0.687
**ART duration**												
≤12 months	1.00				1.00				1.00			
>12 months	2.79	0.51	15.16	0.235	0.91	0.24	3.44	0.885	1.40	0.60	3.26	0.435
**Employment status**												
Un employed	1.00				1.00				1.00			
Employed	0.56	0.12	2.56	0.451	2.35	0.56	6.93	0.296	0.59	0.29	1.20	0.146
**Economic status**												
Low	1.00				1.00				1.00			
High	0.06	0.01	0.36	0.002	0.28	0.08	0.98	0.047	1.33	0.67	2.62	0.410
**Food security**												
Secured	1.00				1.00				1.00			
Insecure	16.24	1.08	244.43	0.044	10.77	1.60	72.54	0.015	1.60	0.57	4.42	0.371
Hunger	15.99	1.70	150.56	0.015	4.55	0.87	23.99	0.074	6.93	3.35	14.33	<0.001

Adjusted for; Age, Sex, Birth weight, Care giver's education level, ART duration, employment status, House hold wealth and Food Security.

### Duration of RUTF intervention and the nutrition status of HIV-positive children

Table 3 shows the result of bivariate association of RUTF duration and nutritional outcome of ART-treated HIV-positive children. Among children who received RUTF (N = 140), about 18% children remained stunted after four or more months of RUTF intervention compared to about 69% of those who received RUTF intervention in less than four months (P < 0.001). None of children who received RUTF for four or more months had either underweight or wasting.

**Table 3:** Bivariate association between RUTF duration and nutrition status

		**<4 months**		≥**4 months**		**P-Value**
**Nutrition status**		**N**	**%**	**N**	**%**	
HAZ (stunting)						
	> − 2SD*	29	51.8	69	82.1	<0.001
	<−2SD**	27	48.2	15	17.9	
WAZ (underweight)						
	> − 2SD*	52	92.9	84	100.0	0.049
	<−2SD**	4	7.1	0	0.0	
WHZ (wasting)						
	> − 2SD*	52	92.9	84	100.0	0.049
	<−2SD**	4	7.1	0	0.0	

* Normal, ** Moderate, or severe undernutrition.

## Discussion

This study found that RUTF treated HIV-positive children under ART had lower proportions of underweight and wasting compared to RUTF naïve HIV-positive children under ART. Furthermore, children treated by RUTF for at least four months had lower proportions of underweight, wasting, and stunting compared to their counterparts. Our study is the first to show the effect of RUTF treatment duration on both acute and chronic undernutrition in the era of ART scaling up.

In this study, about 97% of children treated with RUTF were found to have normal weight-for-age and weight-for-height. This is an important achievement. This is because, all of these children were either severely wasted or underweight or both at the baseline. Compared with RUTF-naïve, the RUTF treated children had a significantly better nutrition status. Such significant achievement of RUTF treatment can be explained as follows. First, RUTF ingredients are energy dense, with lipids and proteins [5], which play an important role to recover the wasted lean tissue [3]. Previous studies also reported the improvement of severe acute undernutrition among ART naïve HIV-positive children treated with RUTF [6,9]. Second, children have an additional advantage of frequent contact with nutrition specialists [36,37], in the presence of nutrition supplement interventions [38-40]. This might improve their undernutrition status through closer monitoring, follow up, and nutritional and hygienic counseling. In a long run, it may also improve their chronic undernutrition status [37,38,41].

While our results showed low wasting and underweight proportions among RUTF treated children compared to RUTF naïve children, proportions of stunting were not significantly different among them. Stunting has many persistent determinants including poverty, food insecurity [42,43], and poor caregivers’ education [44]. To improve this chronic undernutrition, longer treatment duration may be necessary. In our stratified analysis, children who received the treatment for at least four months were less likely to be stunted compared to their counterparts. This may be due to the cumulative effect of the high energy and protein nutrients they obtain from the high energy and protein diet [3], and frequent contact with nutritionist as we have stated above.

Apart from RUTF intervention, this study also found other factors associated with undernutrition among ART treated children. These factors include male sex, low birth weight, lower economic status, and food insecurity. Previous study conducted among ART-treated children in Tanzania [16] and among ART naïve HIV-positive children in South Africa [45] also supports these findings.

Our results should be interpreted with care owing to some limitations. First, the cross-sectional design would limit direction of causal relationship. Using this design, it may be difficult to examine the effect of food in a population that has high food insecurity. In our multivariate analyses, however, we controlled for food insecurity and other factors that were found to be associated with undernutrition in a previous study in the same region [16]. Moreover, the effectiveness of RUTF has also been shown among HIV-negative children [46,47] and ART-naïve HIV-positive children in Sub Saharan Africa [6,7,9]. According to the treatment protocol, all the children in the intervention group had severe underweight or wasting before the treatment initiation. Second, our results may not be generalized beyond the urban settings where this research was conducted. However, these results may be considered in the townships or cities in Tanzania or countries in the region with similar socio-economic characteristics.

In conclusion, among HIV-positive children under ART, provision of RUTF was associated with low proportions of wasting and underweight statuses. Furthermore, RUTF treatment for at least four months was associated with lower proportions of underweight, wasting and stunting. This result suggests that supplementation with RUTF at least for four months has a potential to improve undernutrition among HIV-positive children under ART in the clinical settings. RUTF may be useful in countries like Tanzania and others with high proportion of undernutrition among ART-treated HIV-positive children [16]. It is therefore important to consider scaling up the RUTF intervention in the same pace as ART programs in Tanzania and other countries in the region.

## Abbreviations

AIDS: Acquired immunodeficiency syndrome; AOR: Adjusted odd ratio; ART: Antiretroviral therapy; CI: Confidence interval; CTC: Care and treatment center; ENA: Emergency nutrition assessment; HAZ: Height for age z-score; HFSS: Household food security scale; HIV: Human immunodeficiency virus; PCA: Principal component analysis; RUTF: Ready to use therapeutic foods; TDHS: Tanzania demographic and health survey; WAZ: Weight for age z-score; WHO: World Health Organization; WHZ: Weight for height z-score.

## Competing interests

Authors declare that they have no competing interests.

## Authors’ contributions

BFS conceived the research question, designed the study, conducted the fieldwork, analyzed data, and prepared the manuscript draft. KCP was involved to conceptualize the study and contributed to the study design, statistical analysis, interpretation of the data, and revisions of the manuscript. LBM conducted the fieldwork, data entry, and preparation of the manuscript. KO was involved in the study design. JY was involved in the study design and revision of the manuscript. DPU was involved in logistic preparation for fieldwork, supervision of the fieldwork, and revision of the manuscript. NPM was involved in fieldwork, logistics support, and revision of the manuscript. MJ monitored the study progress, contributed to design of the study, and revised the manuscript. All authors read and approved the final manuscript draft.
